# Supplementary material for: The Dose-Response Associations of Sugar-Sweetened Beverage Intake with the Risk of Stroke, Depression, Cancer, and Cause-Specific Mortality: A Systematic Review and Meta-Analysis of Prospective Studies
Source: Nutrients. 2022 Feb 12;14(4):777. doi: 10.3390/nu14040777 (PMC8875574; doi:10.3390/nu14040777)
Supplement: Supplementary file 1 [file nutrients-14-00777-s001.zip › nutrients-1568967-supplementary.pdf]

Supplementary Files

Appendix:

Table S1. Heterogeneity and Egger's test

Table S2. Sensitivity of stroke

Table S3. Sensitivity of depression

Table S4. Sensitivity of cancer

Table S5. Sensitivity of mortality

Figure S1. Funnel plots for stroke

Figure S2. Funnel plots for depression

Figure S3. Funnel plots for cancers

Figure S4. Funnel plots for mortality

Text S1. Search strategies

Text S2. Unpublished studies

Table S1. Heterogeneity and Egger’s test

	**Stroke**	**Depression**	**Cancer**	**Mortality**
**Heterogeneity** (I^2)	29.9%	0%	0%	68.2%
**Egger’s test** (*p value*)	0.495	0.847	0.01	0.489

Table S2. Sensitivity analysis of stroke.

Stroke	RR	95% Confidence Interval	
Drop Bernstein, 2014 (HPF)			
Bernstein, 2014 (NHS)	1.190	0.965	1.467
Eshak, 2012 (men)	0.760	0.580	0.996
Eshak, 2012 (women)	1.210	0.844	1.734
Larsson, 2014	1.190	1.014	1.396
Gardener, 2012	1.001	0.641	1.562
Pase, 2017	1.220	0.537	2.770
Pacheco, 2020	1.210	1.006	1.456
Total	1.128	1.029	1.236
Drop Bernstein, 2014 (NHS)			
Bernstein, 2014 (HPF)	1.080	0.804	1.451
Eshak, 2012 (men)	0.760	0.580	0.996
Eshak, 2012 (women)	1.210	0.844	1.734
Larsson, 2014	1.190	1.014	1.396
Gardener, 2012	1.001	0.641	1.562
Pase, 2017	1.220	0.537	2.770
Pacheco, 2020	1.210	1.006	1.456
Total	1.110	1.008	1.222
Drop Eshak, 2012 (men)			
Bernstein, 2014 (HPF)	1.080	0.804	1.451
Bernstein, 2014 (NHS)	1.190	0.965	1.467
Eshak, 2012 (women)	1.210	0.844	1.734
Larsson, 2014	1.190	1.014	1.396
Gardener, 2012	1.001	0.641	1.562
Pase, 2017	1.220	0.537	2.770
Pacheco, 2020	1.210	1.006	1.456
Total	1.176	1.073	1.291
Drop Eshak, 2012 (women)			
Bernstein, 2014 (HPF)	1.080	0.804	1.451
Bernstein, 2014 (NHS)	1.190	0.965	1.467
Eshak, 2012 (men)	0.760	0.580	0.996
Larsson, 2014	1.190	1.014	1.396
Gardener, 2012	1.001	0.641	1.562
Pase, 2017	1.220	0.537	2.770
Pacheco, 2020	1.210	1.006	1.456

Total	1.118	1.022	1.224
Drop Larsson, 2014			
Bernstein, 2014 (HPF)	1.080	0.804	1.451
Bernstein, 2014 (NHS)	1.190	0.965	1.467
Eshak, 2012 (men)	0.760	0.580	0.996
Eshak, 2012 (women)	1.210	0.844	1.734
Gardener, 2012	1.001	0.641	1.562
Pase, 2017	1.220	0.537	2.770
Pacheco, 2020	1.210	1.006	1.456
Total	1.096	1.001	1.217
Drop Gardener, 2012			
Bernstein, 2014 (HPF)	1.080	0.804	1.451
Bernstein, 2014 (NHS)	1.190	0.965	1.467
Eshak, 2012 (men)	0.760	0.580	0.996
Eshak, 2012 (women)	1.210	0.844	1.734
Larsson, 2014	1.190	1.014	1.396
Pase, 2017	1.220	0.537	2.770
Pacheco, 2020	1.210	1.006	1.456
Total	1.129	1.032	1.234
Drop Pase, 2017			
Bernstein, 2014 (HPF)	1.080	0.804	1.451
Bernstein, 2014 (NHS)	1.190	0.965	1.467
Eshak, 2012 (men)	0.760	0.580	0.996
Eshak, 2012 (women)	1.210	0.844	1.734
Larsson, 2014	1.190	1.014	1.396
Gardener, 2012	1.001	0.641	1.562
Pacheco, 2020	1.210	1.006	1.456
Total	1.123	1.028	1.226
Drop Pacheco, 2020			
Bernstein, 2014 (HPF)	1.080	0.804	1.451
Bernstein, 2014 (NHS)	1.190	0.965	1.467
Eshak, 2012 (men)	0.760	0.580	0.996
Eshak, 2012 (women)	1.210	0.844	1.734
Larsson, 2014	1.190	1.014	1.396
Gardener, 2012	1.001	0.641	1.562
Pase, 2017	1.220	0.537	2.770
Total	1.100	1.002	1.215

Note: We performed sensitivity analysis by dropping a single study and repeating the meta-analysis.

Table S3. Sensitivity analysis of depression.

Depression	RR	95% Confidence Interval	
Drop Guo, 2014 (men)			
Guo, 2014 (women)	1.250	1.034	1.512
Sanchez-Villegas, 2017	1.120	0.868	1.445
Knüppel, 2017	1.600	0.839	3.050
Total	1.220	1.052	1.415
Drop Guo, 2014 (women)			
Guo, 2014 (men)	1.320	1.081	1.612
Sanchez-Villegas, 2017	1.120	0.868	1.445
Knüppel, 2017	1.600	0.839	3.050
Total	1.258	1.079	1.466
Drop Sanchez-Villegas, 2017			
Guo, 2014 (men)	1.320	1.081	1.612
Guo, 2014 (women)	1.250	1.034	1.512
Knüppel, 2017	1.600	0.839	3.050
Total	1.295	1.132	1.482
Drop Knüppel, 2017			
Guo, 2014 (men)	1.320	1.081	1.612
Guo, 2014 (women)	1.250	1.034	1.512
Sanchez-Villegas, 2017	1.120	0.868	1.445
Total	1.244	1.102	1.404

Note: We performed sensitivity analysis by dropping a single study and repeating the meta-analysis**.**

Table S4. Sensitivity analysis of cancer.

Cancer	RR	95% Confidence Interval	
Drop Bao, 2008			
Bassett, 2020	1.020	0.856	1.215
Chazelas, 2019	1.300	1.091	1.549
Drake, 2012	1.130	0.898	1.422
Holick, 2010	1.230	0.553	2.737
Inoue-Choi, 2013	1.740	0.999	3.031
Larsson, 2006	2.300	0.636	8.314
Makarem, 2018	1.000	0.787	1.271
Mueller, 2010	1.870	0.648	5.397
Nomura, 2016	1.030	0.927	1.144
Nothlings, 2007	1.070	0.797	1.437
Pacheco, 2019	1.140	0.815	1.594
Romanos-Nanclares, 2019	1.410	0.573	3.468
Schernhammer, 2005	1.130	0.781	1.636
Stepien, 2016	1.460	0.813	2.621
Total	1.099	1.029	1.174
Drop Bassett, 2020			
Bao, 2008	1.070	0.850	1.347
Chazelas, 2019	1.300	1.091	1.549
Drake, 2012	1.130	0.898	1.422
Holick,2010	1.230	0.553	2.737
Inoue-Choi, 2013	1.740	0.999	3.031
Larsson, 2006	2.300	0.636	8.314
Makarem, 2018	1.000	0.787	1.271
Mueller, 2010	1.870	0.648	5.397
Nomura, 2016	1.030	0.927	1.144
Nothlings, 2007	1.070	0.797	1.437
Pacheco, 2019	1.140	0.815	1.594
Romanos-Nanclares, 2019	1.410	0.573	3.468
Schernhammer, 2005	1.130	0.781	1.636
Stepien, 2016	1.460	0.813	2.621
Total	1.109	1.036	1.187
Drop Chazelas, 2019			
Bao, 2008	1.070	0.850	1.347
Bassett, 2020	1.020	0.856	1.215

Drake, 2012	1.130	0.898	1.422
Holick,2010	1.230	0.553	2.737
Inoue-Choi, 2013	1.740	0.999	3.031
Larsson, 2006	2.300	0.636	8.314
Makarem, 2018	1.000	0.787	1.271
Mueller, 2010	1.870	0.648	5.397
Nomura, 2016	1.030	0.927	1.144
Nothlings, 2007	1.070	0.797	1.437
Pacheco, 2019	1.140	0.815	1.594
Romanos-Nanclares, 2019	1.410	0.573	3.468
Schernhammer, 2005	1.130	0.781	1.636
Stepien, 2016	1.460	0.813	2.621
Total	1.069	1.001	1.144
Drop Drake, 2012			
Bao, 2008	1.070	0.850	1.347
Bassett, 2020	1.020	0.856	1.215
Chazelas, 2019	1.300	1.091	1.549
Holick,2010	1.230	0.553	2.737
Inoue-Choi, 2013	1.740	0.999	3.031
Larsson, 2006	2.300	0.636	8.314
Makarem, 2018	1.000	0.787	1.271
Mueller, 2010	1.870	0.648	5.397
Nomura, 2016	1.030	0.927	1.144
Nothlings, 2007	1.070	0.797	1.437
Pacheco, 2019	1.140	0.815	1.594
Romanos-Nanclares, 2019	1.410	0.573	3.468
Schernhammer, 2005	1.130	0.781	1.636
Stepien, 2016	1.460	0.813	2.621
Total	1.094	1.024	1.169
Drop Holick,2010			
Bao, 2008	1.070	0.850	1.347
Bassett, 2020	1.020	0.856	1.215
Chazelas, 2019	1.300	1.091	1.549
Drake, 2012	1.130	0.898	1.422
Inoue-Choi, 2013	1.740	0.999	3.031
Larsson, 2006	2.300	0.636	8.314
Makarem, 2018	1.000	0.787	1.271

Mueller, 2010	1.870	0.648	5.397
Nomura, 2016	1.030	0.927	1.144
Nothlings, 2007	1.070	0.797	1.437
Pacheco, 2019	1.140	0.815	1.594
Romanos-Nanclares, 2019	1.410	0.573	3.468
Schernhammer, 2005	1.130	0.781	1.636
Stepien, 2016	1.460	0.813	2.621
Total	1.096	1.029	1.168
Drop Inoue-Choi, 2013			
Bao, 2008	1.070	0.850	1.347
Bassett, 2020	1.020	0.856	1.215
Chazelas, 2019	1.300	1.091	1.549
Drake, 2012	1.130	0.898	1.422
Holick,2010	1.230	0.553	2.737
Larsson, 2006	2.300	0.636	8.314
Makarem, 2018	1.000	0.787	1.271
Mueller, 2010	1.870	0.648	5.397
Nomura, 2016	1.030	0.927	1.144
Nothlings, 2007	1.070	0.797	1.437
Pacheco, 2019	1.140	0.815	1.594
Romanos-Nanclares, 2019	1.410	0.573	3.468
Schernhammer, 2005	1.130	0.781	1.636
Stepien, 2016	1.460	0.813	2.621
Total	1.090	1.023	1.162
Drop Larsson, 2006			
Bao, 2008	1.070	0.850	1.347
Bassett, 2020	1.020	0.856	1.215
Chazelas, 2019	1.300	1.091	1.549
Drake, 2012	1.130	0.898	1.422
Holick,2010	1.230	0.553	2.737
Inoue-Choi, 2013	1.740	0.999	3.031
Makarem, 2018	1.000	0.787	1.271
Mueller, 2010	1.870	0.648	5.397
Nomura, 2016	1.030	0.927	1.144
Nothlings, 2007	1.070	0.797	1.437
Pacheco, 2019	1.140	0.815	1.594
Romanos-Nanclares, 2019	1.410	0.573	3.468

Schernhammer, 2005	1.130	0.781	1.636
Stepien, 2016	1.460	0.813	2.621
Total	1.095	1.028	1.166
Drop Makarem, 2018			
Bao, 2008	1.070	0.850	1.347
Bassett, 2020	1.020	0.856	1.215
Chazelas, 2019	1.300	1.091	1.549
Drake, 2012	1.130	0.898	1.422
Holick,2010	1.230	0.553	2.737
Inoue-Choi, 2013	1.740	0.999	3.031
Larsson, 2006	2.300	0.636	8.314
Mueller, 2010	1.870	0.648	5.397
Nomura, 2016	1.030	0.927	1.144
Nothlings, 2007	1.070	0.797	1.437
Pacheco, 2019	1.140	0.815	1.594
Romanos-Nanclares, 2019	1.410	0.573	3.468
Schernhammer, 2005	1.130	0.781	1.636
Stepien, 2016	1.460	0.813	2.621
Total	1.104	1.034	1.179
Drop Mueller, 2010			
Bao, 2008	1.070	0.850	1.347
Bassett, 2020	1.020	0.856	1.215
Chazelas, 2019	1.300	1.091	1.549
Drake, 2012	1.130	0.898	1.422
Holick,2010	1.230	0.553	2.737
Inoue-Choi, 2013	1.740	0.999	3.031
Larsson, 2006	2.300	0.636	8.314
Makarem, 2018	1.000	0.787	1.271
Nomura, 2016	1.030	0.927	1.144
Nothlings, 2007	1.070	0.797	1.437
Pacheco, 2019	1.140	0.815	1.594
Romanos-Nanclares, 2019	1.410	0.573	3.468
Schernhammer, 2005	1.130	0.781	1.636
Stepien, 2016	1.460	0.813	2.621
Total	1.095	1.027	1.166
Drop Nomura, 2016			

Bao, 2008	1.070	0.850	1.347
Bassett, 2020	1.020	0.856	1.215
Chazelas, 2019	1.300	1.091	1.549
Drake, 2012	1.130	0.898	1.422
Holick,2010	1.230	0.553	2.737
Inoue-Choi, 2013	1.740	0.999	3.031
Larsson, 2006	2.300	0.636	8.314
Makarem, 2018	1.000	0.787	1.271
Mueller, 2010	1.870	0.648	5.397
Nothlings, 2007	1.070	0.797	1.437
Pacheco, 2019	1.140	0.815	1.594
Romanos-Nanclares, 2019	1.410	0.573	3.468
Schernhammer, 2005	1.130	0.781	1.636
Stepien, 2016	1.460	0.813	2.621
Total	1.137	1.050	1.231
Drop Nothlings, 2007			
Bao, 2008	1.070	0.850	1.347
Bassett, 2020	1.020	0.856	1.215
Chazelas, 2019	1.300	1.091	1.549
Drake, 2012	1.130	0.898	1.422
Holick,2010	1.230	0.553	2.737
Inoue-Choi, 2013	1.740	0.999	3.031
Larsson, 2006	2.300	0.636	8.314
Makarem, 2018	1.000	0.787	1.271
Mueller, 2010	1.870	0.648	5.397
Nomura, 2016	1.030	0.927	1.144
Pacheco, 2019	1.140	0.815	1.594
Romanos-Nanclares, 2019	1.410	0.573	3.468
Schernhammer, 2005	1.130	0.781	1.636
Stepien, 2016	1.460	0.813	2.621
Total	1.098	1.029	1.172
Drop Pacheco, 2019			
Bao, 2008	1.070	0.850	1.347
Bassett, 2020	1.020	0.856	1.215
Chazelas, 2019	1.300	1.091	1.549
Drake, 2012	1.130	0.898	1.422
Holick,2010	1.230	0.553	2.737

Inoue-Choi, 2013	1.740	0.999	3.031
Larsson, 2006	2.300	0.636	8.314
Makarem, 2018	1.000	0.787	1.271
Mueller, 2010	1.870	0.648	5.397
Nomura, 2016	1.030	0.927	1.144
Nothlings, 2007	1.070	0.797	1.437
Romanos-Nanclares, 2019	1.410	0.573	3.468
Schernhammer, 2005	1.130	0.781	1.636
Stepien, 2016	1.460	0.813	2.621
Total	1.095	1.027	1.168
Drop Romanos-Nanclares, 2019			
Bao, 2008	1.070	0.850	1.347
Bassett, 2020	1.020	0.856	1.215
Chazelas, 2019	1.300	1.091	1.549
Drake, 2012	1.130	0.898	1.422
Holick,2010	1.230	0.553	2.737
Inoue-Choi, 2013	1.740	0.999	3.031
Larsson, 2006	2.300	0.636	8.314
Makarem, 2018	1.000	0.787	1.271
Mueller, 2010	1.870	0.648	5.397
Nomura, 2016	1.030	0.927	1.144
Nothlings, 2007	1.070	0.797	1.437
Pacheco, 2019	1.140	0.815	1.594
Schernhammer, 2005	1.130	0.781	1.636
Stepien, 2016	1.460	0.813	2.621
Total	1.095	1.028	1.167
Drop Schernhammer, 2005			
Bao, 2008	1.070	0.850	1.347
Bassett, 2020	1.020	0.856	1.215
Chazelas, 2019	1.300	1.091	1.549
Drake, 2012	1.130	0.898	1.422
Holick,2010	1.230	0.553	2.737
Inoue-Choi, 2013	1.740	0.999	3.031
Larsson, 2006	2.300	0.636	8.314
Makarem, 2018	1.000	0.787	1.271
Mueller, 2010	1.870	0.648	5.397
Nomura, 2016	1.030	0.927	1.144

Nothlings, 2007	1.070	0.797	1.437
Pacheco, 2019	1.140	0.815	1.594
Romanos-Nanclares, 2019	1.410	0.573	3.468
Stepien, 2016	1.460	0.813	2.621
Total	1.096	1.028	1.169
Drop Stepien, 2016			
Bao, 2008	1.070	0.850	1.347
Bassett, 2020	1.020	0.856	1.215
Chazelas, 2019	1.300	1.091	1.549
Drake, 2012	1.130	0.898	1.422
Holick,2010	1.230	0.553	2.737
Inoue-Choi, 2013	1.740	0.999	3.031
Larsson, 2006	2.300	0.636	8.314
Makarem, 2018	1.000	0.787	1.271
Mueller, 2010	1.870	0.648	5.397
Nomura, 2016	1.030	0.927	1.144
Nothlings, 2007	1.070	0.797	1.437
Pacheco, 2019	1.140	0.815	1.594
Romanos-Nanclares, 2019	1.410	0.573	3.468
Schernhammer, 2005	1.130	0.781	1.636
Total	1.093	1.026	1.165

Note: We performed sensitivity analysis by dropping a single study and repeating the meta-analysis.

Table S5. Sensitivity analysis of mortality.

Mortality	RR	95% Confidence Interval	
Drop Anderson, 2020			
Barrington, 2016	1.190	1.066	1.328
Collin, 2019	1.140	0.952	1.365
Malik, 2019 (women)	1.250	1.142	1.368
Malik, 2019 (men)	1.120	0.984	1.275
Mullee, 2019	1.080	1.002	1.164
Odegaard, 2015	0.960	0.877	1.050
Paganini-Hill, 2007	1.030	0.914	1.161
Zhang, 2020	1.050	1.009	1.093
Total	1.075	1.046	1.106
Drop Barrington, 2016			
Anderson, 2020	1.840	1.144	2.959
Collin, 2019	1.140	0.952	1.365
Malik, 2019 (women)	1.250	1.142	1.368
Malik, 2019 (men)	1.120	0.984	1.275
Mullee, 2019	1.080	1.002	1.164
Odegaard, 2015	0.960	0.877	1.050
Paganini-Hill, 2007	1.030	0.914	1.161
Zhang, 2020	1.050	1.009	1.093
Total	1.070	1.040	1.101
Drop Collin, 2019			
Anderson, 2020	1.840	1.144	2.959
Barrington, 2016	1.190	1.066	1.328
Malik, 2019 (women)	1.250	1.142	1.368
Malik, 2019 (men)	1.120	0.984	1.275
Mullee, 2019	1.080	1.002	1.164
Odegaard, 2015	0.960	0.877	1.050
Paganini-Hill, 2007	1.030	0.914	1.161
Zhang, 2020	1.050	1.009	1.093
Total	1.076	1.046	1.107
Drop Malik, 2019 (women)			
Anderson, 2020	1.840	1.144	2.959
Barrington, 2016	1.190	1.066	1.328
Collin, 2019	1.140	0.952	1.365
Malik, 2019 (men)	1.120	0.984	1.275

Mullee, 2019	1.080	1.002	1.164
Odegaard, 2015	0.960	0.877	1.050
Paganini-Hill, 2007	1.030	0.914	1.161
Zhang, 2020	1.050	1.009	1.093
Total	1.061	1.030	1.092
Drop Malik, 2019 (men)			
Anderson, 2020	1.840	1.144	2.959
Barrington, 2016	1.190	1.066	1.328
Collin, 2019	1.140	0.952	1.365
Malik, 2019 (women)	1.250	1.142	1.368
Mullee, 2019	1.080	1.002	1.164
Odegaard, 2015	0.960	0.877	1.050
Paganini-Hill, 2007	1.030	0.914	1.161
Zhang, 2020	1.050	1.009	1.093
Total	1.075	1.045	1.106
Drop Mullee, 2019			
Anderson, 2020	1.840	1.144	2.959
Barrington, 2016	1.190	1.066	1.328
Collin, 2019	1.140	0.952	1.365
Malik, 2019 (women)	1.250	1.142	1.368
Malik, 2019 (men)	1.120	0.984	1.275
Odegaard, 2015	0.960	0.877	1.050
Paganini-Hill, 2007	1.030	0.914	1.161
Zhang, 2020	1.050	1.009	1.093
Total	1.077	1.045	1.110
Drop Odegaard, 2015			
Anderson, 2020	1.840	1.144	2.959
Barrington, 2016	1.190	1.066	1.328
Collin, 2019	1.140	0.952	1.365
Malik, 2019 (women)	1.250	1.142	1.368
Malik, 2019 (men)	1.120	0.984	1.275
Mullee, 2019	1.080	1.002	1.164
Paganini-Hill, 2007	1.030	0.914	1.161
Zhang, 2020	1.050	1.009	1.093
Total	1.091	1.059	1.123
Drop Paganini-Hill, 2007			
Anderson, 2020	1.840	1.144	2.959
Barrington, 2016	1.190	1.066	1.328

Collin, 2019	1.140	0.952	1.365
Malik, 2019 (women)	1.250	1.142	1.368
Malik, 2019 (men)	1.120	0.984	1.275
Mullee, 2019	1.080	1.002	1.164
Odegaard, 2015	0.960	0.877	1.050
Zhang, 2020	1.050	1.009	1.093
Total	1.080	1.050	1.111
Drop Zhang, 2020			
Anderson, 2020	1.840	1.144	2.959
Barrington, 2016	1.190	1.066	1.328
Collin, 2019	1.140	0.952	1.365
Malik, 2019 (women)	1.250	1.142	1.368
Malik, 2019 (men)	1.120	0.984	1.275
Mullee, 2019	1.080	1.002	1.164
Odegaard, 2015	0.960	0.877	1.050
Paganini-Hill, 2007	1.030	0.914	1.161
Total	1.103	1.062	1.147

Note: We performed sensitivity analysis by dropping a single study and repeating the meta-analysis.

Figure S1. Funnel plots for stroke

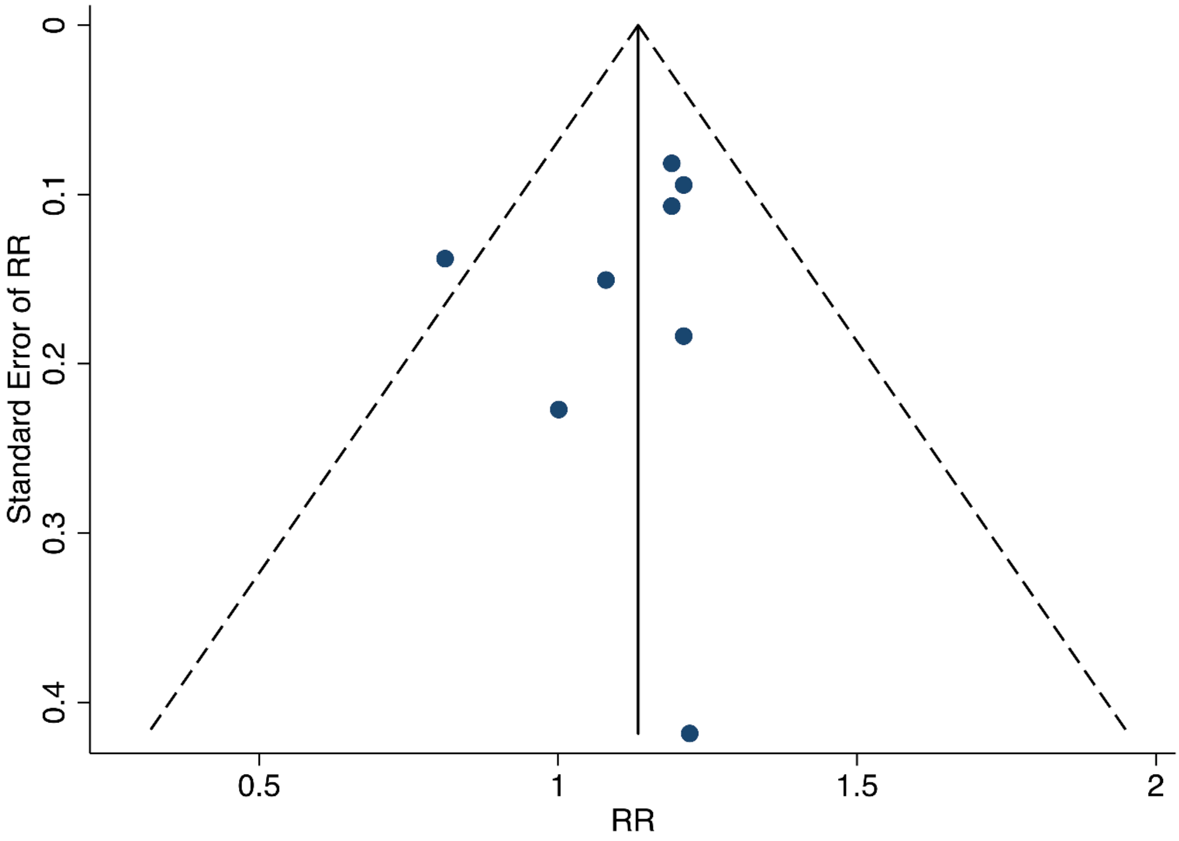

Figure S2. Funnel plots for depression

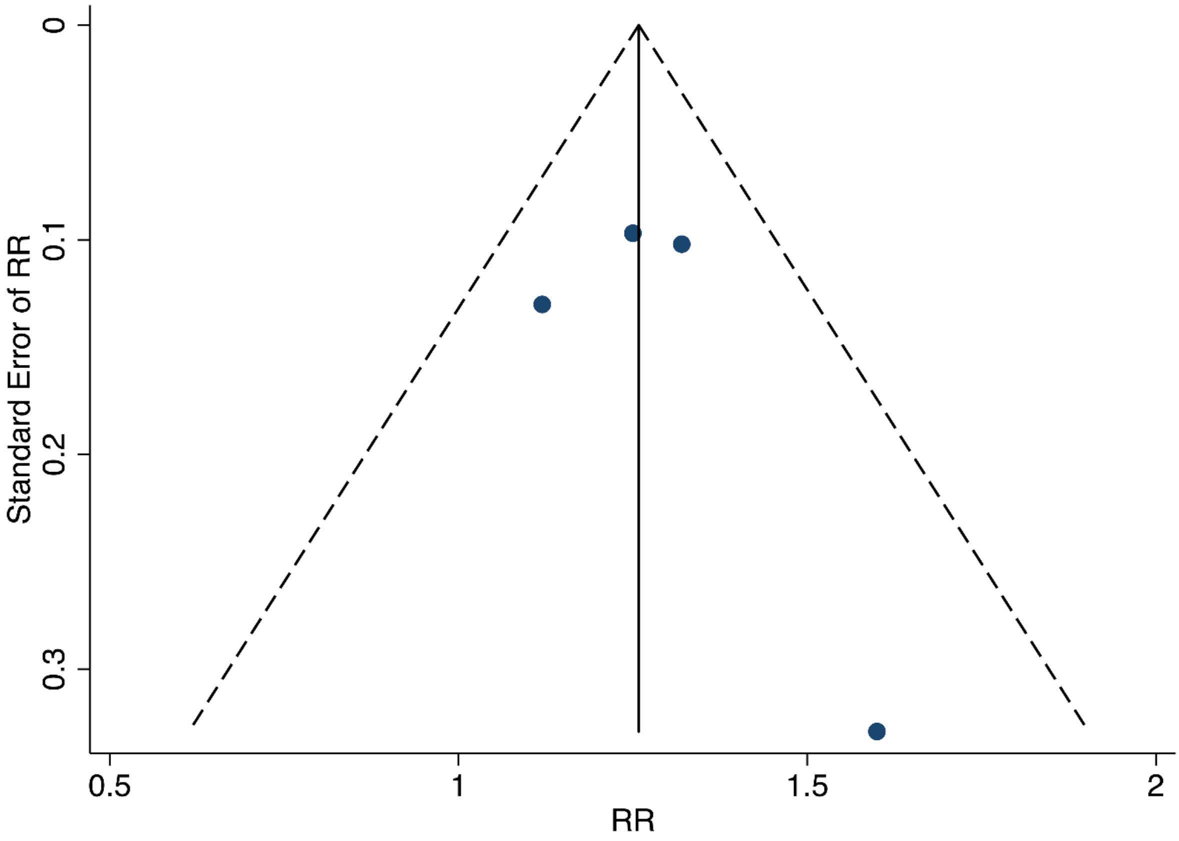

Figure S3. Funnel plots for cancers

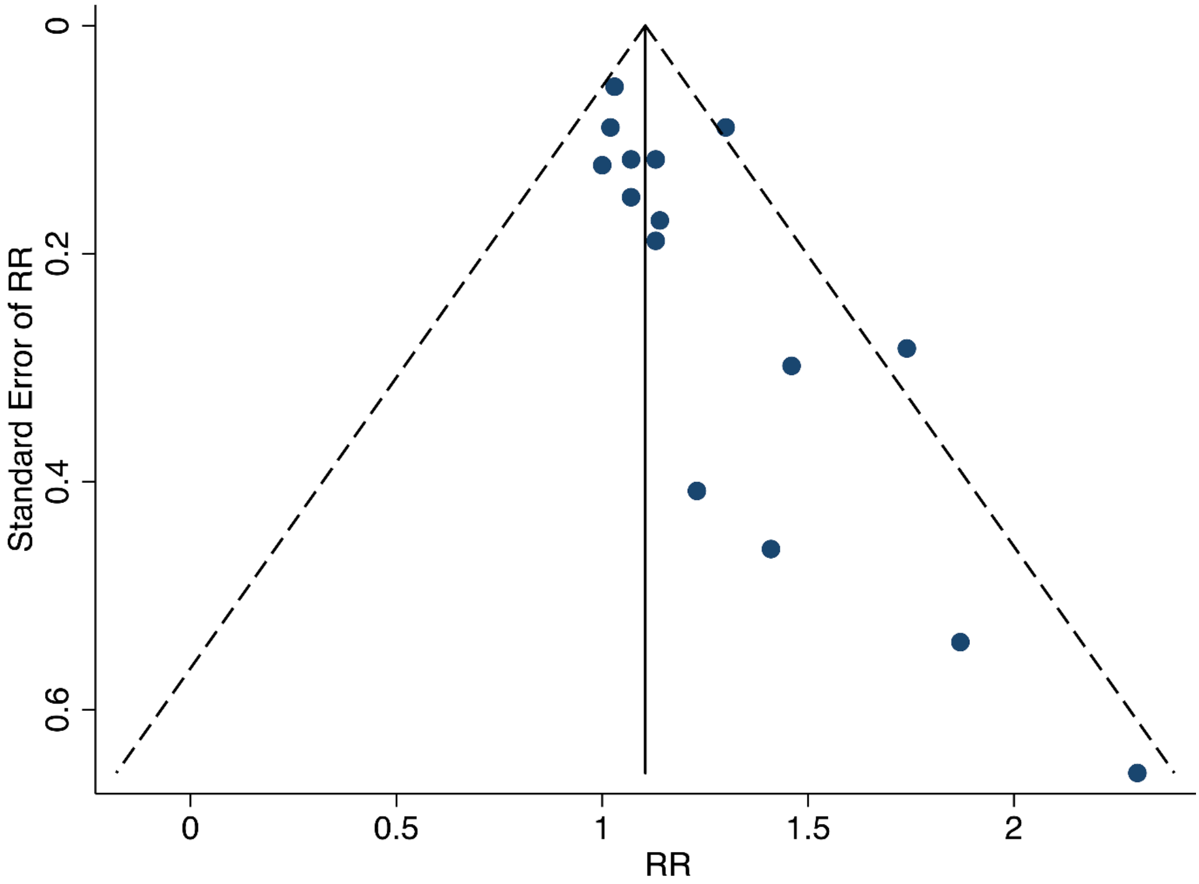

Figure S4. Funnel plots for mortality

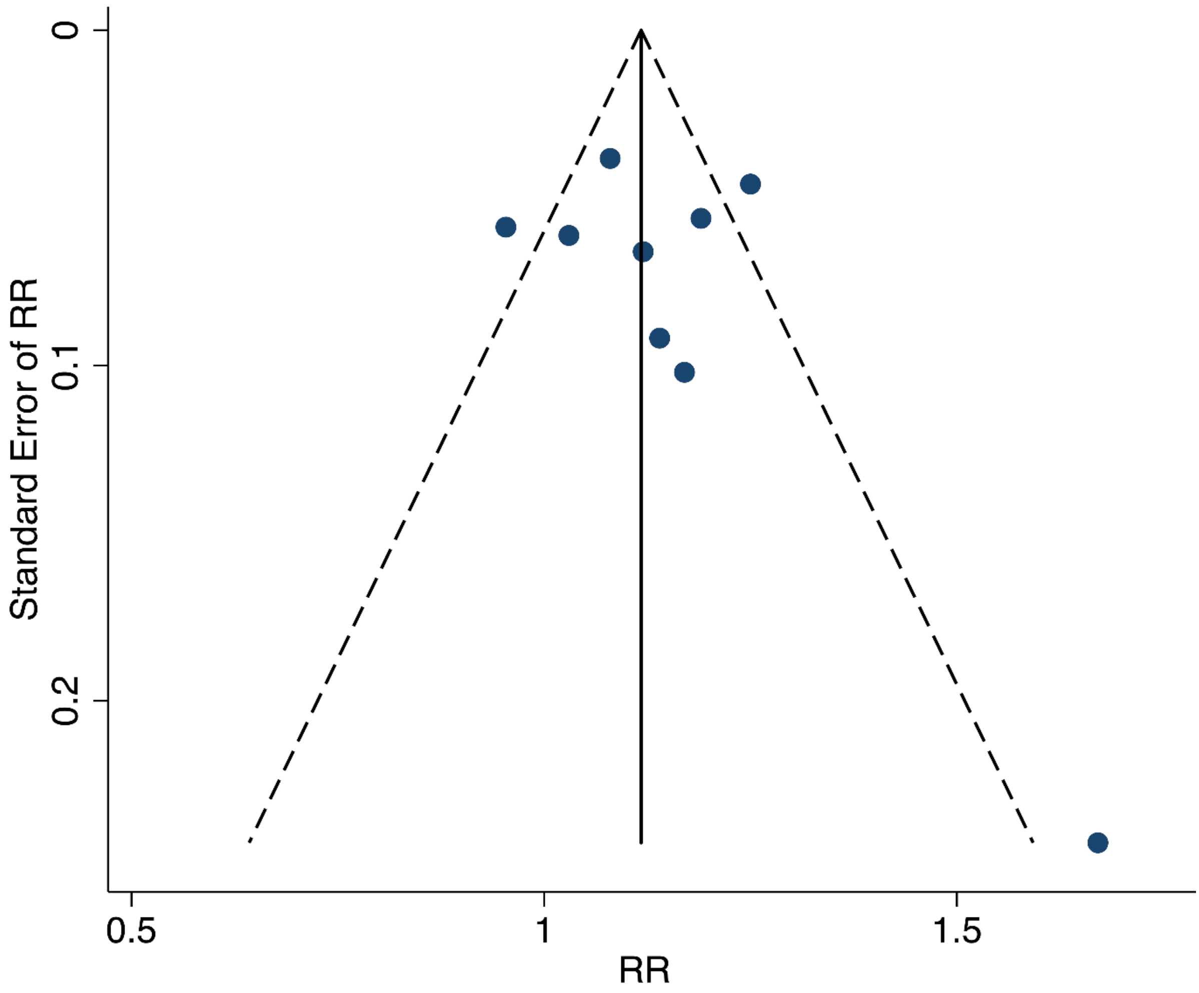

Text S1. Search strategies

PubMed: ((soda[Title/Abstract] OR pop[Title/Abstract] OR juic*[Title/Abstract] OR drin*[Title/Abstract] OR beverag*[Title/Abstract] OR sweetened[Title/Abstract]) AND (stoke[Title/Abstract] OR depression[Title/Abstract] OR cance*[Title/Abstract] OR mortalit*[Title/Abstract] OR deat*[Title/Abstract] OR fatal[Title/Abstract] OR surviv*[Title/Abstract])) AND (prospective[Title/Abstract] OR longitudinal[Title/Abstract] OR cohor*[Title/Abstract] OR follow-up[Title/Abstract]) in abstract and title; 5241 hits on Nov 10, 2021

Cochrane Library: (soda OR pop OR juic* OR drin* OR beverage* OR sweetened in Title) AND (stoke OR depression OR cance* OR mortalit* OR deat* OR fatal OR surviv* in Title) AND (prospective OR longitudinal OR cohor* OR follow-up) in Title Abstract Keyword; 1341 hits on Nov 10, 2021

Embase: (("soda" OR "pop" OR "juice" OR "drink" OR "drinks" OR "beverage" OR "beverages" OR "sweetened") and ("stoke" OR "depression" OR "cancer" OR "mortality" OR "death" OR "fatal" OR "survival") and ("prospective" OR "longitudinal" OR "cohort" OR "follow-up")) in abstract and title; 1099 hits on Nov 10, 2021

Web of Science: Title=("soda" OR "pop" OR "juic*" OR "drin*" OR "beverag*" OR "sweetened") AND Title =("stoke" OR "depression" OR "cance*" OR "mortalit*" OR "deat*" OR "fatal" OR "surviv*") AND Title =("prospective" OR "longitudinal" OR "cohor*" OR "follow-up") in abstract and title; 1158 hits on Nov 10, 2021

Text S2. Unpublished studies

No.	Recruitment status	Prospective Registration	Main ID	Public Title	Date of Registration
1	Recruiting	No	NCT04710108	Testing message modality of culturally appropriate nutrition communication for Mexican American women	12/01/2021
2	Not Recruiting	No	ACTRN12620000605998	Associations between diet quality and common mental disorders in emerging adults: results from a nationally representative sample	25/05/2020
3	Not Recruiting	Yes	NL8628	The effect of information and pricing interventions for meat on food purchases in a virtual supermarket	18/05/2020
4	Recruiting	No	ChiCTR1900020781	Intervention of restriction on sugar-sweetened beverage consumption among students by health promotion ecological model	2019-01-19
5	Not recruiting	Yes	NCT03543644	Strategies to oppose sugars with non-nutritive sweeteners or water (stop sugars now) trial	02/05/2018
6	Recruiting	Yes	NCT03527277	Orange juice and sugar intervention study	10/04/2018
7	Not recruiting	Yes	NCT02947802	Sugar sweetened beverage labeling support	26/10/2016
8	Not recruiting	No	NCT02926001	Evaluation of the impact of a sugar-sweetened beverage tax in Chile	04/10/2016
9	Not Recruiting	Yes	ISRCTN14964130	Does the bottle size for cola drinks influence how much people drink at home? A feasibility and acceptability study	18/05/2015
10	Recruiting	No	ACTRN12621000259842	Feasibility and acceptability of a digital educational physical activity program for childhood cancer survivors	10/03/2021
11	Not Recruiting	Yes	NL8628	The effect of information and pricing interventions for meat on food purchases in a virtual supermarket	18/05/2020

Note: Studies identified by searching *the WHO International Clinical Trials Registry Platform (ICTRP) search portal* and *ClinicalTrials.gov*, accessed Nov 10, 2021
